# Serum calprotectin as a marker of neonatal sepsis: a hospital-based cross-sectional diagnostic study

**DOI:** 10.12688/f1000research.132099.1

**Published:** 2023-06-07

**Authors:** Pardha Ramineni, Sowmini Padmanabh Kamath, Poornima Manjrekar, Padmanabh Kamath, Prasanna Mithra, Vaman Kulkarni

**Affiliations:** 1 Department of Pediatrics, Kasturba Medical College, Mangalore, Manipal Academy of Higher Education, Manipal, India; 2 Department of Biochemistry, Kasturba Medical College, Mangalore, Manipal Academy of Higher Education, Manipal, India; 3 Department of Cardiology, Kasturba Medical College, Mangalore, Manipal Academy of Higher Education, Manipal, India; 4 Department of Community Medicine, Kasturba Medical College, Mangalore, Manipal Academy of Higher Education, Manipal, India; 5 Department of Community Medicine and Family Medicine, All India Institute of Medical Sciences (AIIMS), Bibinagar, Telangana, India

**Keywords:** biomarkers, blood culture, Calprotectin, neonatal sepsis, newborn, Procalcitonin, ROC curve, sepsis

## Abstract

**Background:** Despite significant advances in neonatal care, neonatal sepsis remains a major contributor to mortality, morbidity, and protracted hospitalization. The development of early possible diagnostic indicators for newborn sepsis is critical. Since calprotectin participates in major biological processes, it could be a diagnostic marker for infection/inflammation. This study aimed to estimate serum calprotectin in neonates with clinical sepsis. In addition, we compared serum calprotectin with standard sepsis markers and serum procalcitonin to evaluate its diagnostic accuracy.

**Methods:** A hospital-based cross-sectional diagnostic study of neonates identified with clinical sepsis using standard criteria was carried out. We compared estimated serum calprotectin levels to serum procalcitonin levels and conventional sepsis markers (leucocyte count, blood culture, immature to total neutrophil ratio, and C- reactive protein). We used SPSS version 25 to analyze the data. To examine diagnostic accuracy and determine a cut-off value for serum calprotectin, we used the receiver operating characteristics (ROC) curve.

**Results:** Of the 83 subjects included, 36.5% (30/83) had blood culture positive status, the median value of serum calprotectin being 0.93 ng/ml (0.67 to 1.3). Respiratory, cardiovascular, and gastrointestinal instabilities were present in 67.5% (56/83), 59% (49/83), and 50.1% (42/83) cases, respectively. The presence of positive and negative blood cultures did not significantly affect sepsis parameters (p=0.09). On ROC, calprotectin was not predictive for blood culture positivity (sensitivity: 50%; specificity: 44% at 0.83 ng/ml of serum calprotectin) and C-reactive protein (CRP) levels (sensitivity: 57%; specificity: 67% at serum calprotectin levels of 0.89 ng/ml). However, compared with serum procalcitonin, serum calprotectin at 1.2 ng/ml had sensitivity and specificity of 60% and 73%, respectively.

**Conclusions:** Serum calprotectin did not show a distinct advantage over the existing sepsis markers. Serum calprotectin level at 1.2 ng/ml had a sensitivity and specificity of 60% and 73%, respectively, compared to serum procalcitonin in detecting neonatal sepsis.

## Introduction

Sepsis is a severe and potentially lethal organ dysfunction often produced by an inadequate host response to an infection. ^1^ Neonates are a unique cohort of populations showing differences in physiology and immunology between children and adults. Even though the last decade has shown a substantial reduction in neonatal mortality globally, septicemia continues to be a significant contributor, accounting for 11 million neonatal deaths yearly. ^2^ In neonates the onset of sepsis is often quiet with minimal unclear and nonspecific signs. An accurate and early diagnosis plays a critical role. It is for this purpose that a host of novel biomarkers are being explored. The most significant aspect is that they are markers of adaptive immunological responses that are not well established during the initial post-natal period. The gold standard for organism isolation remains to be blood culture. Nevertheless, the culture results are only available after 48 hours and, sometimes, fail to show the growth of microbes despite the clear clinical picture of sepsis. Hence, neonatal sepsis remains challenging for clinicians to ensure accurate diagnosis at the appropriate time.

Calprotectin, also known as MRP 8/14, S100A8/S100A9, is a zinc and calcium-binding protein heterodimer. It is primarily located in the cytosolic neutrophil fraction and comprises nearly 30-40% of the protein content. ^3^ It is released into the circulation due to exocytosis of granules from activated neutrophils. ^4^ Calprotectin intracellular roles include activation of neutrophilic NADPH oxidase and cytoskeletal regulation of phagocyte migration. ^5^^,^^6^ It contains apoptosis-inducing, antibacterial, proinflammatory, and oxidant-scavenging properties. ^7^^,^^8^ *In vitro* studies have demonstrated bacteriostatic, fungi-static, and resistance to enzymatic degradation. Calprotectin elevation in extracellular fluids of inflammatory disorders such as abscesses, cystic fibrosis, and rheumatoid arthritis have been reported. ^9^^–^^12^

Calprotectin is secreted into circulation by innate immune system cells immediately following a host-pathogen interaction. An enzyme-linked immunosorbent assay (ELISA) test can detect it. Its potential use in the diagnosis of various inflammatory diseases is being investigated. Earlier studies have shown different cut-off levels, varying sensitivity, and specificity of serum calprotectin in detecting sepsis in neonates. ^13^^–^^16^ The literature on its diagnostic value in newborn sepsis in the Indian context is, however, scarce.

Procalcitonin is one of the most widely studied markers in sepsis. Monocytes and hepatocytes produce procalcitonin, which rises within four hours and has a half-life between 25 and 30 hours. ^17^ It is thus a reliable indicator of sepsis compared to conventional markers.

According to a comprehensive review and meta-analysis by Vouloumanou *et al.*, procalcitonin has a pooled sensitivity and specificity of 81% and 79% in identifying newborn sepsis. ^18^ According to a recent meta-analysis by Ruan *et al.*, procalcitonin paired with C-reactive protein (CRP) or presepsin was more accurate in diagnosing newborn sepsis. They also stated the various cut-off levels used to define neonatal sepsis ranged from 0.5 to 2.2 ng/ml. ^19^ Procalcitonin levels increase physiologically in the first 24 hours of life, with elevations additionally seen in non-infectious causes such as trauma, surgery, and respiratory distress syndrome. These confounding factors limit the utility of procalcitonin in neonatal sepsis, specifically in early-onset sepsis. ^15^

This study aimed to estimate calprotectin levels in newborns with clinical sepsis and compare its diagnostic accuracy with other sepsis markers such as blood culture, CRP, and serum procalcitonin.

## Methods

### Study setting

We conducted cross-sectional diagnostic research at Tertiary Neonatal Critical Care Units linked to Kasturba Medical College, Mangalore, Manipal Academy of Higher Education, Manipal, India. We included admitted neonates with clinical sepsis diagnosed by clinical criteria ^20^ between December 2018 to September 2020 by convenient sampling.

### Study design

**Figure 1.**
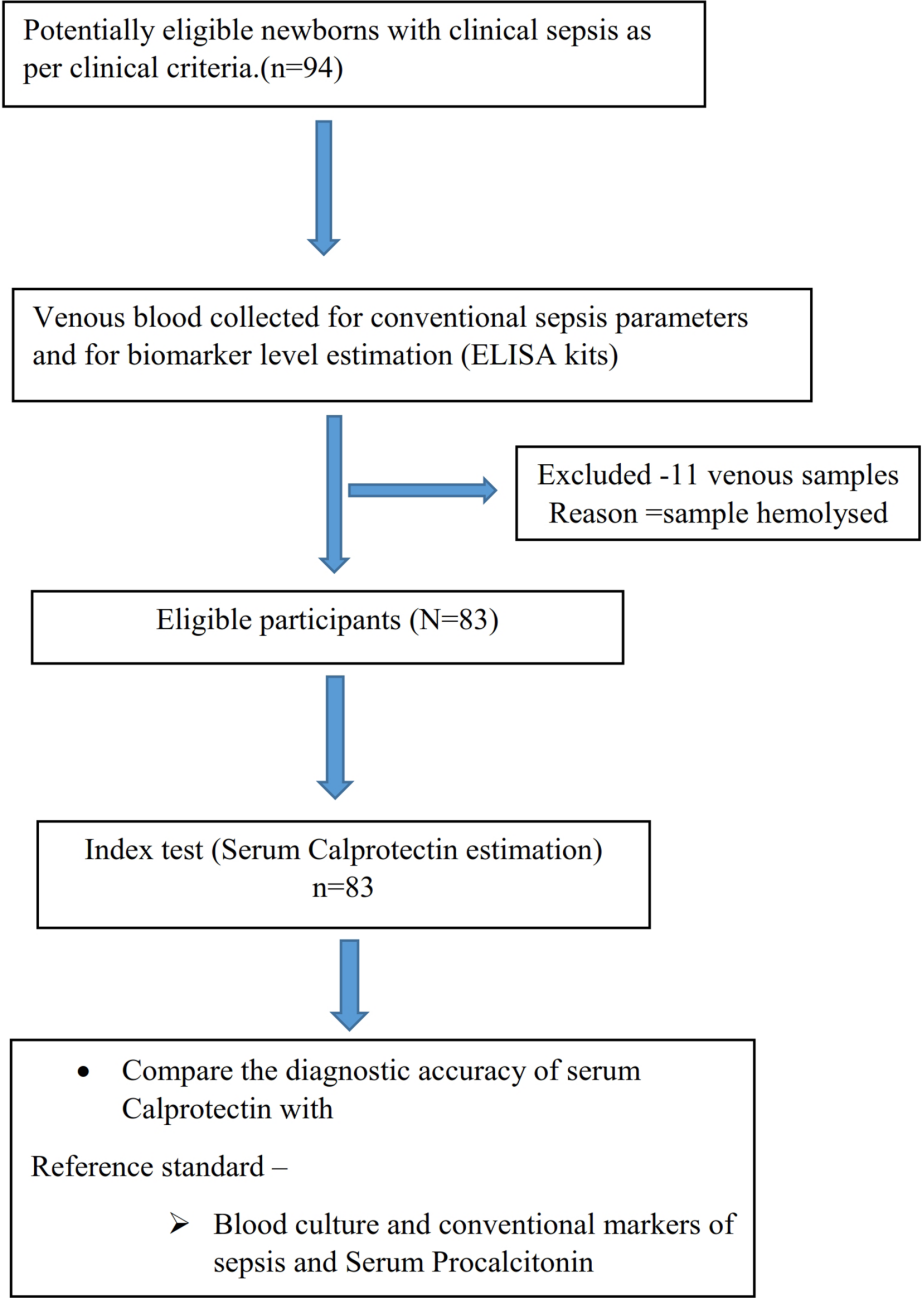
Study flow diagram.

This research follows the Standards for Reporting Diagnostic Accuracy (STARD) ^21^ statement guidelines. The reporting guidelines contain a completed STARD checklist. ^22^ Figure 1 depicts the study flow according to STARD criteria. ^22^

### Sample size

Based on a prior study by Terrin *et al.*, ^13^ where the sensitivity of serum calprotectin levels in predicting neonatal sepsis was 89% and using a normogram with 10% absolute precision, 95% confidence interval, with 10% non-responsive rate, the sample size was estimated to be 77 and rounded off to 80.

### Ethics and data collection

The 1964 Declaration of Helsinki and its later amendments, as well as other related ethical principles, were followed in the conduct of the study. The Institutional ethics committee of Kasturba Medical College, Mangalore (IEC KMC MLR 10-18/378, dated 17/10/2018) approved the study, and we obtained appropriate hospital authorities' permits. We approached the parents and guardians of newborns who met the inclusion criteria and informed them in their native tongue about the study's goals. We gave the parents a participant information sheet with answers to the most frequently asked queries (as in *Extended data*). ^22^ We obtained signed informed consent (as in *Extended data*) ^22^ if the parents or guardians were willing for their newborns to participate. We gathered the baseline demographic information and neonatal medical history using a validated semi-structured pretested proforma (as in *Extended data*). ^22^

### Inclusion and exclusion criteria

Admitted neonates with clinical sepsis diagnosed as per the clinical criteria were included. We excluded ventilated neonates with respiratory or circulatory failure, previous exposure to antibiotics, preterm less than 32 weeks, had APGAR scores less than "3" or had conditions such as persistent pulmonary hypertension of newborn, congenital malformations, surgical-related disorders, and severe intracranial bleeding.

### Operational definitions

Clinical sepsis in newborns was defined as the presence of two or more of the following characteristics, namely respiratory instability, cardiovascular instability, gastrointestinal instability, temperature instability, sclerema or petechial rash, and nonspecific features. ^20^ We considered sepsis screen to be positive if two or more of the following observed laboratory parameters, namely CRP >6 mg/dl, total leucocyte count (TLC) >20,000×10 ^9^/L or <4,000×10 ^9^/L and immature to total neutrophil ratio (I/T ratio) >0.2. ^20^ The sex of the neonate as male or female was determined by external examination of body characteristics by the investigator.

### Sample collection

We collected a venous blood sample to estimate TLC, I/T ratio, CRP, blood culture, serum procalcitonin, and serum calprotectin levels. Beckman Coulter's automated system and Nephelometry calculated the total leucocyte count and CRP, respectively. A peripheral smear examination differentiated the leucocytes, and we determined the I/T ratio. For blood cultures, we collected about 1 mL of venous blood under aseptic conditions. We inoculated blood into blood culture medium bottles (BD BACTEC™ PedsPlus™/F culture vials) before being evaluated for growth at regular intervals.

For serum procalcitonin and serum calprotectin levels estimation, we collected an aliquot of 2 ml venous blood in plain tubes, centrifuged it at 5,000 rotations per minute for 15 minutes, and kept the separated serum at -80°C until further processing. Serum calprotectin levels were estimated using the Human CALP (Calprotectin) ELISA Kit (Catalog no: ELK4602 **)** from ELK Biotechnology ^®^, China, on LISA plus ELISA reader. The detection range for the calprotectin kit was between 31.25 and 2,000 pg/ml, and its sensitivity was 13.7 pg/ml with high specificity. Serum procalcitonin was estimated using the Human PCT (Procalcitonin) ELISA Kit (Catalog No: E-EL-H1492) from Elabscience ^®^, China, on LISA plus ELISA reader. The procalcitonin kit exhibited a sensitivity of 18.75 pg/ml, a detection range of 31.25 to 2,000 pg/ml, and a coefficient of variation under 10%. We converted the measured values of serum calprotectin and procalcitonin into ng/ml since the conventional expression of serum procalcitonin is in ng/ml.

### Statistical analysis

We used IBM SPSS Statistics (RRID:SCR_016479) for Windows, Version 25.0 (IBM Corp., Armonk, NY) to analyze the data. We expressed patient characteristics in proportions. We calculated the median and interquartile ranges for the following parameters: TLC, CRP, I/T ratio, serum calprotectin levels, and procalcitonin levels. By using Mann-Whitney U test, we compared the values of serum calprotectin and other conventional markers of sepsis among culture-positive and culture-negative sepsis. We performed a receiver operator characteristic (ROC) curve to obtain a cut-off for serum calprotectin levels to detect sepsis in neonates  when compared with the conventional sepsis parameters/serum procalcitonin.

## Results

Of the 83 neonates included, 63% (52/83) were male, 79.5% (66/83) were inborn babies, and 54% (45/83) were delivered by cesarean section. The median gestational age and birth weight values were 261 (IQR: 244–274) days and 2,100 (IQR: 1,600–2,800) grams, respectively. The median length of stay in the NICU was 6 (IQR: 5–11) days. The median values of CRP, TLC, serum calprotectin, and serum procalcitonin are presented in Table 1. ^22^ There was no significant association between neonatal sex and median values of serum calprotectin and procalcitonin levels (p values of 0.43 and 0.75, respectively).

**Table 1.** Median values of septic screening parameters among neonates with clinical sepsis.

Septic parameters	Median	Inter Quartile Range (25–75th)
C-Reactive protein (mg/dl)	27.09	12.09–43.56
Total Leucocyte Count (cells/mm ^3^)	11,200	3,900–17,200
I/T ratio	0.18	0.12–0.22
Serum Calprotectin Level (ng/ml)	0.93	0.67–1.3
Serum Procalcitonin Level (ng/ml)	0.14	0.03–0.27

**Figure 2.**
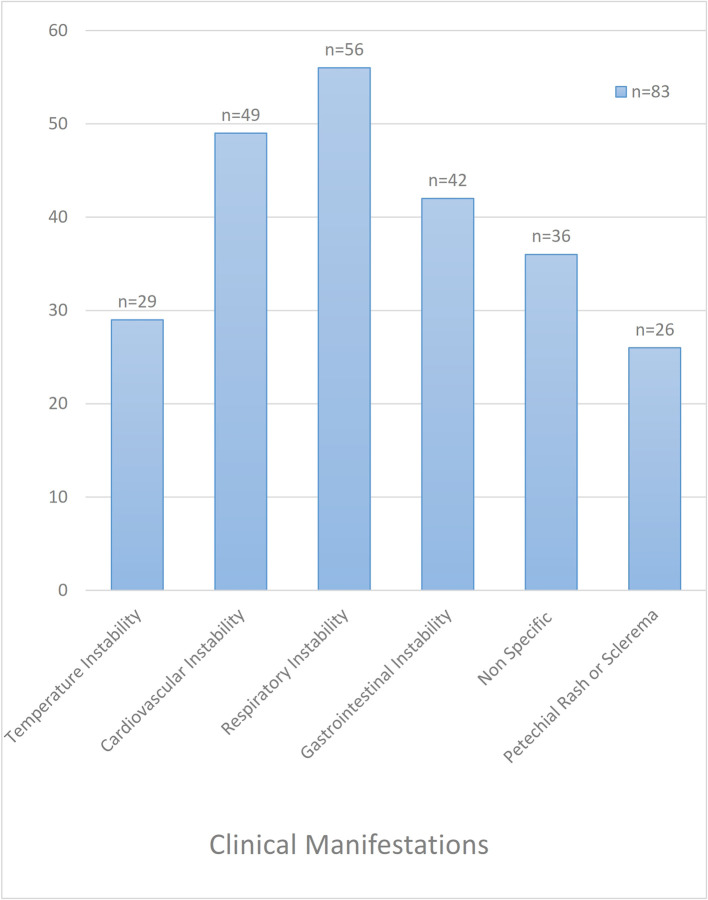
Clinical presentations of neonates with clinical sepsis.

Among the clinical criteria for suspecting clinical sepsis in neonates ( Figure 2), respiratory instability was present in 68% (56/83), cardiovascular and gastrointestinal instability in 59% (49/83) and 51% (42/83) cases, respectively. While petechial rash or sclerema was visible in 16% (26/83) of newborns, temperature instability (hypo/hyperthermia) was identified in 19% (29/83) of cases.

Increased oxygen requirement, tachypnea, and apnea were found in 41% (34/83), 36.1% (30/83), and 16.8% (14/83) cases, respectively. Impaired peripheral perfusion was seen in 45% (37/83) of neonates and was the most common clinical sign of sepsis noted in this study. Tachycardia, hypotension, and skin mottling were seen in 21.6% (18/83), 9.6% (8/83), and 20.4% (17/83) neonates, respectively. Poor feeding was the most common gastrointestinal presentation noted in 28.9% (24/83) neonates, followed by feed intolerance and abdominal distension in 22.8% (19/83) and 9.6% (8/83) cases, respectively.

Blood culture was positive in 36.1% (30/83) of newborns. Among the 30 culture-positive cases, we found bacterial growth in 76% (23/30) and fungal sepsis ( *Candida* species) in 24% (7/30) of newborns. Among the bacterial sepsis, Klebsiella and Methicillin-Resistant *Staphylococcus aureus* (MRSA) were isolated in six cases each, followed by *Acinetobacter* in four patients. Further, we documented *Citrobacter* and *Pseudomonas* growth in three cases each and Methicillin-sensitive *Staphylococcus aureus* in one case.

Table 2 compares the median serum calprotectin, procalcitonin levels, and conventional markers of sepsis among the blood culture-positive and culture-negative cases. The differences in the median values of serum calprotectin, procalcitonin, TLC, and I/T ratio between blood culture-positive and culture-negative groups were not statistically significant.

**Table 2.** Comparative characteristics between culture positive and culture negative sepsis.

Septic parameter	Blood Culture Positive Sepsis Median (IQR)	Blood culture Negative Sepsis Median (IQR)	P value
C-Reactive protein (mg/dl)	28.18 (11.24–44.31)	25.19 (12.51–42.62)	0.63
Total Leucocyte Count (cells/mm ^3^)	10,950 (3,850–15,700)	11,200 (3,900–18,600)	0.45
I/T ratio	0.18 (0.14–0.22)	0.18 (0.10–0.23)	0.74
Serum Calprotectin Level (ng/ml)	1.14 (0.69–1.37)	0.89 (0.67–1.25)	0.41
Serum Procalcitonin Level (ng/ml)	0.13 (0.03–0.23)	0.18 (0.03–0.31)	0.41

**Figure 3.**
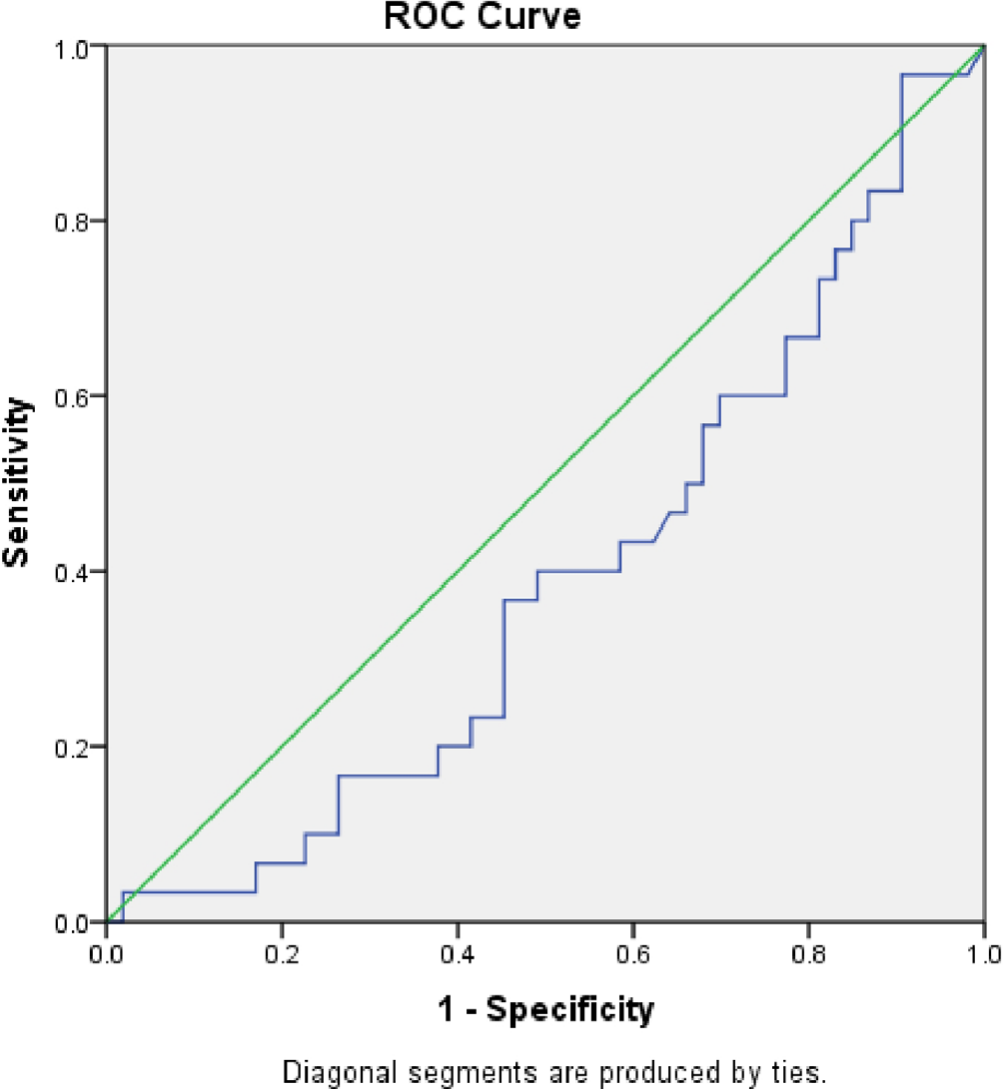
Receiver Operator Characteristic (ROC) comparing accuracy of serum calprotectin with blood culture.

**Figure 4.**
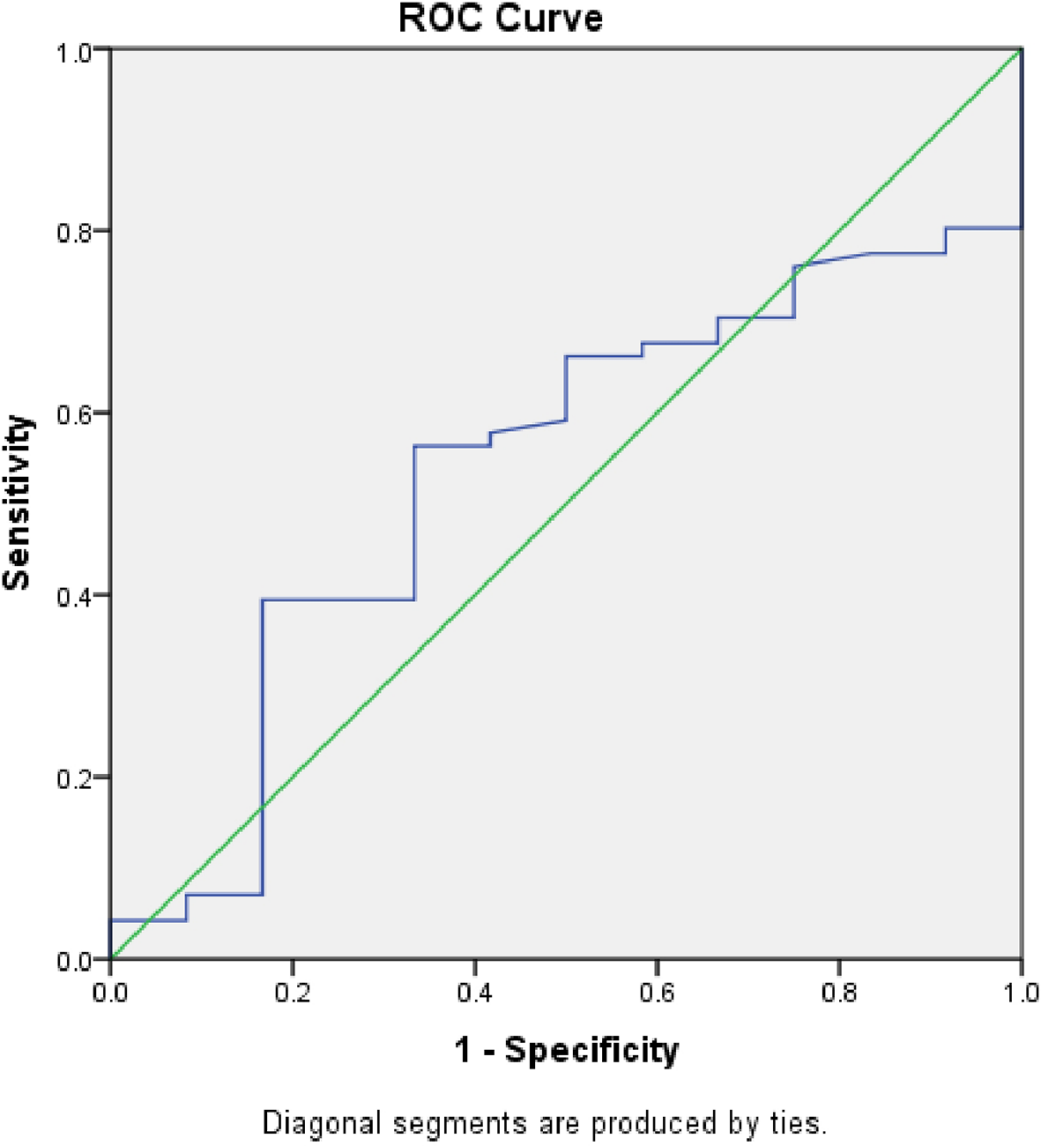
Receiver Operator Characteristic (ROC) comparing accuracy of serum calprotectin with C-reactive protein.

The area under the curve (AUC) was 0.39 (S.E. 0.06, p=0.1, CI=0.27 to 0.51) when serum calprotectin was compared to blood culture using a ROC curve ( Figure 3). We determined the sensitivity and specificity to be 50% and 44%, respectively, at a cut-off level of 0.83 ng/ml. The serum calprotectin ROC curve exhibited an AUC of 0.536 (S.E. 0.08, p=0.69, CI=0.37 to 0.70) compared to CRP ( Figure 4). We discovered the sensitivity and specificity to be 57% and 67%, respectively, at a cut-off level of 0.89 ng/ml of calprotectin.

**Figure 5.**
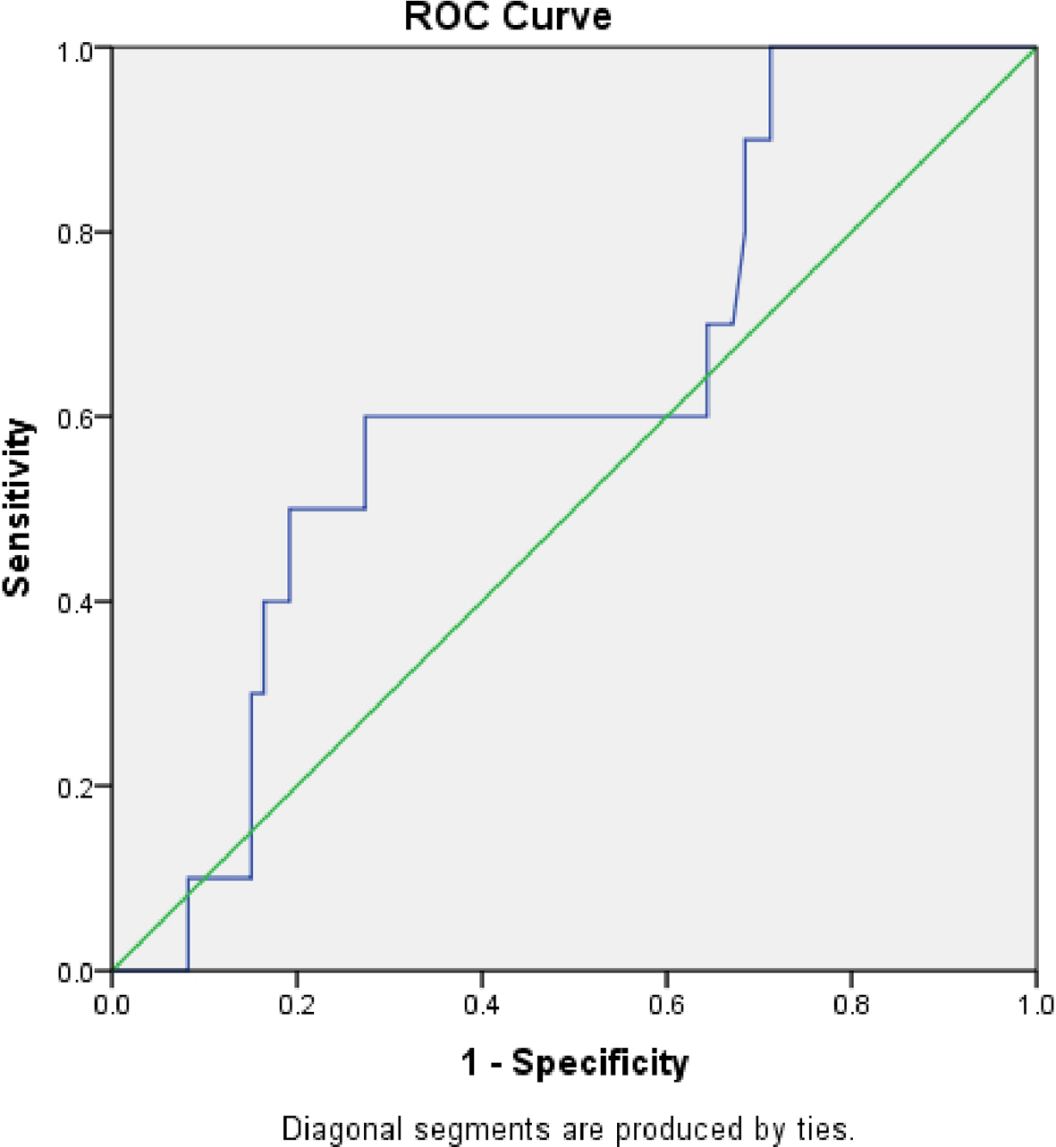
Receiver Operator Characteristic (ROC) comparing accuracy of serum calprotectin with serum procalcitonin.

Figure 5 displays the ROC curve contrasting serum calprotectin levels and procalcitonin. The AUC was 0.627 (S.E. 0.09, p=0.20, CI=0.45 to 0.80). We determined the sensitivity and specificity to be 60% and 73% at a 1.2 ng/ml cut-off level of serum calprotectin.

## Discussion

Calprotectin is an innate immune marker; thus, we investigated its significance as a biological marker for the early diagnosis of newborn septicemia. We found the median serum calprotectin levels in clinically septic neonates to be 0.93 ng/ml (IQR 0.67–1.3). Earlier studies documented the median or mean serum calprotectin levels in septic, non-septic, and control neonates. ^13^^–^^16^^,^^23^ The varied broad range of reported values may be because of the differences in the kit employed for estimation.

We observed that the serum calprotectin was higher in the blood culture positive group as compared to the culture negative group, however the difference in the current study was not statistically significant. Similar to this, previous studies found higher calprotectin levels in blood culture positive groups compared to negative groups, ^14^^–^^16^^,^^23^ with statistically significant differences. ^13^^,^^23^ In addition, in contrast with previous research, we were unable to determine the precise reason why our study cohort had lower median serum calprotectin levels. Possible explanations include earlier studies evaluated serum calprotectin in particular neonatal groups, namely very low birth weight ^13^ and late-onset sepsis. ^23^ The current study, however, comprised a broad sample of newborns with early and late-onset sepsis and a range of birth weights, offering a unique perspective that calls for further subgroup research. Furthermore, the kits used in various studies were different.

Respiratory instability was the most common manifestation seen in more than two thirds of cases (68%) in our study and was similar to the survey by Attia *et al.* ^15^ In the current study, calprotectin levels did not significantly differ between males and females, which is consistent with other research. ^15^^,^^23^

Blood culture-positive cases contributed to 36.5% of the neonates in our research and are in line with the expected culture-positivity rates of 10 to 30% in neonatal sepsis. ^24^ Previous studies have documented blood culture-positive cases in 19.5% ^14^, 16.6% ^23^ and 75% ^15^ cases. The most common isolates in the present study were *Klebsiella* and MRSA and were concurrent with the published literature. ^14^^,^^15^

When compared with CRP, blood culture, and serum procalcitonin levels, the ROC curves generated sensitivity and specificity levels with cut-off values of serum calprotectin to detect neonatal sepsis. In the current study, serum calprotectin had a sensitivity of 60% and specificity of 73% compared to serum procalcitonin in identifying newborn sepsis at a 1.2 ng/ml cut-off. In the literature, limited data compare serum calprotectin with serum procalcitonin levels in neonates. However, our study showed poor sensitivity and specificity concerning the gold standard blood culture. The correlation of serum calprotectin with blood culture was not documented in previous studies. ^13^^–^^16^^,^^23^

Serum calprotectin had sensitivity between 42.5–92% and specificity between 70–96% with cut-off levels between 1.4 to 38.3 μg/ml, as per earlier studies. ^13^^–^^16^^,^^23^ The wide variations in the values may be due to different kits and a lack of international standards. It is unclear how our serum calprotectin levels were lower than that documented in previous studies. Possibilities of varying kit specifics and influences from the diverse neonatal population may be contributory, this being the first Indian neonatal population studied.

This study's limitations were the lack of a control group for comparison, the relatively smaller sample size, and the sensitivity and detection range of the ELISA kit used to estimate serum calprotectin. Further multicenter studies involving a larger population of neonates are warranted given broad ranges of mean/median values of serum calprotectin, varied ranges of sensitivity and specificity with different cut-off values, and due to varying usages of kits in various studies.

## Conclusions

Serum calprotectin is not superior to existing sepsis markers. Serum calprotectin levels equal to and above 1.2 ng/ml had a sensitivity of 60% and specificity of 73% compared to serum procalcitonin in detecting sepsis in our neonatal population.

## Data Availability

Open Scientific Framework: Serum Calprotectin as a marker of neonatal sepsis – a hospital-based cross-sectional diagnostic study. https://doi.org/10.17605/OSF.IO/6V84E. ^22^ This dataset contains the following underlying data:
• Data excel sheet F1000 research.xlsx • Data Code Key F1000 research.docx Open Scientific Framework: Serum Calprotectin as a marker of neonatal sepsis – a hospital-based cross-sectional diagnostic study. https://doi.org/10.17605/OSF.IO/6V84E. ^22^ This dataset contains the following underlying extended data:
• Parent information sheet.docx • Informed consent form.docx • Study Proforma.docx Open Scientific Framework: STARD checklist for ‘Serum calprotectin as a marker of neonatal sepsis – a hospital-based cross-sectional diagnostic study’. https://doi.org/10.17605/OSF.IO/6V84E. ^22^ Data are available under the terms of the Creative Commons Attribution 4.0 International license (CC-BY 4.0).
