# Supplementary material for: Serum calprotectin as a marker of neonatal sepsis: a hospital-based cross-sectional diagnostic study
Source: F1000Res. 2023 Nov 9;12:626. Originally published 2023 Jun 7. [Version 2] doi: 10.12688/f1000research.132099.2 (PMC10432886; doi:10.12688/f1000research.132099.2)
Supplement: Peer review history

# Reviewer report: Reviewer response for version 1

**DOI:** 10.5256/f1000research.145000.r178896

**Published:** 2023-08-16

**Authors:** Kalyan Chakravarthy Konda

**Affiliations:** 1 Department of Neonatology, Rainbow Children's Hospital, Hyderabad, Telangana, India

Neonatal sepsis remains a significant contributor to morbidity and mortality despite many advances. I firstly congratulate the authors for choosing a rational topic and conducting a well-structured study. The robust methodology deserves appreciation. I would like to recommend a few modifications.

Results section:

1. More preferable to document gestational age in weeks over days.
2. It is surprising to see that sepsis is predominant in the inborn population compared to the outborn. It would be better if there is more elaboration on the base line characteristics of the population - to categorise the population into preterm-term, EOS-LOS, day of investigation, and clinical background (risk factors for sepsis-like PPROM). It helps to understand the study population better and helps to gauge both internal and external validity.
3. Does the comparison between neonatal sex and septic markers have a significant rationale? If not the author may consider not mentioning it. To the best of my knowledge, none of the septic biomarkers have sex-specific significance.
4. Calprotectin has also been explored as a marker of NEC. It is therefore important mentioning any episode of NEC in the study population (especially in the culture-positive group), as it is a bias. Consider adding a subgroup analysis of the marker in the population with GI symptoms and in those without GI symptoms. 

Discussion section:

1. Please avoid repeating the statement about the "lower serum calprotectin levels noticed in the study population and possible explanation of heterogenicity" multiple times in the discussion. 

Is the work clearly and accurately presented and does it cite the current literature?

Yes

If applicable, is the statistical analysis and its interpretation appropriate?

Yes

Are all the source data underlying the results available to ensure full reproducibility?

Yes

Is the study design appropriate and is the work technically sound?

Yes

Are the conclusions drawn adequately supported by the results?

Yes

Are sufficient details of methods and analysis provided to allow replication by others?

Yes

Reviewer Expertise:

Neonatology, Ventilation, POCUS

I confirm that I have read this submission and believe that I have an appropriate level of expertise to confirm that it is of an acceptable scientific standard.

# Response

**Published:** 2023-10-30

**Authors:** Sowmini Padmanabh Kamath

**Affiliations:** Kasturba Medical College, Mangalore, India

**Neonatal sepsis remains a significant contributor to morbidity and mortality despite many advances. I firstly congratulate the authors for choosing a rational topic and conducting a well-structured study. The robust methodology deserves appreciation. I would like to recommend a few modifications.**

**Results section:**

**1. More preferable to document gestational age in weeks over days**.

 A). Yes, we have represented gestational age as weeks now

**2. It is surprising to see that sepsis is predominant in the inborn population compared to the outborn. It would be better if there is more elaboration on the base line characteristics of the population - to categorise the population into preterm-term, EOS-LOS, day of investigation, and clinical background (risk factors for sepsis-like PPROM). It helps to understand the study population better and helps to gauge both internal and external validity.**

**A). **Among 83 neonates, 32 were term AGA, 15 term SGA, 13 Preterm AGA, 21 Preterm SGA, and 2 preterm LGA. Sepsis was early onset (<72 hours) in 36 and late-onset (>72 hours) in 49 babies. The predominant risk factor for sepsis was preterm premature rupture of membranes (PPROM) in 15, prolonged rupture of membranes (PROM) in 6, and maternal urinary tract infections (UTI) in 3 cases.

**3. Does the comparison between neonatal sex and septic markers have a significant rationale? If not the author may consider not mentioning it. To the best of my knowledge, none of the septic biomarkers have sex-specific significance.**

 A). we have made the necessary changes and removed the sentences.

**4. Calprotectin has also been explored as a marker of NEC. It is therefore important mentioning any episode of NEC in the study population (especially in the culture-positive group), as it is a bias. Consider adding a subgroup analysis of the marker in the population with GI symptoms and in those without GI symptoms.**

 A). any episode of NEC in the study population was an exclusion criterion to avoid bias and was included in surgical conditions. Currently, we have specified it as -surgical conditions, including NEC (in the inclusion and exclusion criteria).

 Subgroup analysis of the presence or absence of GI symptoms in neonates did not show any association with biomarkers. The median (IQR) serum calprotectin levels in neonates with the presence and absence of GI symptoms were 0.9 (0.68-1.24) and 0.95(0.67-1.33), respectively, the difference was not statistically significant (p=0.58).

**Discussion section:**

**1. Please avoid repeating the statement about the "lower serum calprotectin levels noticed in the study population and possible explanation of heterogenicity" multiple times in the discussion.**

 A). we have made the necessary changes as suggested.

# Reviewer report: Reviewer response for version 1

**DOI:** 10.5256/f1000research.145000.r178894

**Published:** 2023-06-26

**Authors:** Rathika Damodara Shenoy

**Affiliations:** 1 Department of Paediatric Surgery, K.S. Hegde Medical Academy, NITTE (Deemed to be University), Mangalore, Karnataka, India

Newborn sepsis often presents with non-specific symptoms and signs. Blood cultures are positive in just about one-third. There is a constant search for a sensitive and specific biomarker, and this study has investigated the suitability of serum calprotectin. The comparison has been made against culture-positive sepsis, elevated CRP and procalcitonin. The methodology is well written. The cut-off of PCT used to determine ROC in the study can be added either under Operational definitions or under Statistical analysis sections.

 The median birth weight of the study population of 83 newborns is 2100g. This suggests that the study population was comprised largely of a small gestation-age newborns. It is surprising to note the type of bacterial isolates (Acinetobacter, Citrobacter) and Candida growth (7 of 30) without exposure to prior antibiotics (exclusion criteria).

 The authors can add the factors influencing serum calprotectin levels - is the serum calprotectin response the same in small and appropriate for age newborns, bacterial vs fungal sepsis, if literature is available. In the discussion, it is mentioned that the calprotectin response was similar in male and female newborns, but this is not presented in the Results. This may be deleted from the discussion.

Is the work clearly and accurately presented and does it cite the current literature?

Yes

If applicable, is the statistical analysis and its interpretation appropriate?

Yes

Are all the source data underlying the results available to ensure full reproducibility?

Yes

Is the study design appropriate and is the work technically sound?

Yes

Are the conclusions drawn adequately supported by the results?

Yes

Are sufficient details of methods and analysis provided to allow replication by others?

Yes

Reviewer Expertise:

Genetics & Metabolic disorders

I confirm that I have read this submission and believe that I have an appropriate level of expertise to confirm that it is of an acceptable scientific standard.

# Response

**Published:** 2023-10-30

**Authors:** Sowmini Padmanabh Kamath

**Affiliations:** Kasturba Medical College, Mangalore, India

**Newborn sepsis often presents with non-specific symptoms and signs. Blood cultures are positive in just about one-third. There is a constant search for a sensitive and specific biomarker, and this study has investigated the suitability of serum calprotectin. The comparison has been made against culture-positive sepsis, elevated CRP and procalcitonin. The methodology is well written. The cut-off of PCT used to determine ROC in the study can be added either under Operational definitions or under Statistical analysis sections.**

 A) ****A cut-off of 0.5 ng/ml of PCT was used for the ROC and has been included in the statistical analysis section

**The median birth weight of the study population of 83 newborns is 2100g. This suggests that the study population was comprised largely of a small gestation-age newborns. It is surprising to note the type of bacterial isolates (Acinetobacter, Citrobacter) and Candida growth (7 of 30) without exposure to prior antibiotics (exclusion criteria).**

 A) Median (IQR) gestational age was 37.4 (34.9-39.14) weeks. There were 47 term babies (term AGA: 32, term SGA: 15) and 36 preterm babies (preterm AGA: 13, preterm SGA: 21, and preterm LGA: 2) (have been included in the results section).

 We conducted the study in a district tertiary maternity hospital catering to services in and around the Dakshina Kannada district. Many deliveries occur daily with a high probability of developing drug-resistant strains in the hospital environment. The study population primarily comprised SGA babies (term SGA: 15 and preterm SGA: 21).

 Yes, it is surprising to note the type of bacterial isolates without antibiotic exposure. Similar to our study, Terrin et al. documented 52 culture-positive proven sepsis in neonates, with six neonates showing growth of candida. These rare Acinetobacter, Citrobacter, and Candida growths were predominantly present in preterm SGA neonates and a single-term SGA baby (included in the discussion section).

 We found risk factors such as preterm SGA babies, birth weight less than 1500 grams, intravascular/umbilical catheterization, and NICU stay greater than seven days were prone to have culture-proven sepsis in our study, similar to previous research by Shete et al., and Arora et al.(included in the  discussion section)   

**The authors can add the factors influencing serum calprotectin levels - is the serum calprotectin response the same in small and appropriate for age newborns, bacterial vs fungal sepsis, if literature is available. In the discussion, it is mentioned that the calprotectin response was similar in male and female newborns, but this is not presented in the Results. This may be deleted from the discussion.**

 A). The median (IQR) serum calprotectin levels in term and preterm neonates were 1(0.67-1.33) and 0.85(0.67-1.23), respectively, and this was not statistically significant (p=0.49). Similarly, we found no significant difference in the response of serum calprotectin levels between SGA and AGA neonates and with bacterial versus fungal sepsis (has been included in the results section)

 The literature search does not document the serum calprotectin responses in SGA versus AGA neonates or term versus preterm babies. An earlier article by Bartakova et al., demonstrated the serum concentration of calprotectin to be higher in bacterial sepsis when compared to viral infections; however, limited literature is available on serum calprotectin responses between bacterial versus fungal sepsis (has been included in the discussion section)

 We have deleted the sentence of calprotectin response similar in males and females (discussion section)
